# ENIGMA *CHEK2*gether Project: A Comprehensive Study Identifies Functionally Impaired *CHEK2* Germline Missense Variants Associated with Increased Breast Cancer Risk

**DOI:** 10.1158/1078-0432.CCR-23-0212

**Published:** 2023-07-13

**Authors:** Lenka Stolarova, Petra Kleiblova, Petra Zemankova, Barbora Stastna, Marketa Janatova, Jana Soukupova, Maria Isabel Achatz, Christine Ambrosone, Paraskevi Apostolou, Banu K. Arun, Paul Auer, Mollie Barnard, Birgitte Bertelsen, Biobank Japan, Koichi Matsuda, Yoichiro Kamatani, Takayuki Morisaki, Akiko Nagai, Kaori Muto, Yoshinori Murakami, Yoichi Furukawa, Yuji Yamanashi, Yusuke Nakamura, Taisei Mushiroda, Yukihide Momozawa, Toshihiro Tanaka, Yozo Ohnishi, Michiaki Kubo, Shinichi Higashiue, Shuzo Kobayashi, Shiro Minami, Hiroki Yamaguhci, Hajime Arai, Ken Yamaji, Yasushi Okazaki, Satoshi Asai, Yasuo Takahashi, Tomoaki Fujioka, Wataru Obara, Seijiro Mori, Shigeo Murayama, Satoshi Nagayama, Yoshio Miki, Akihide Masumoto, Akira Yamada, Yasuko Nishizawa, Masahiko Higashiyama, Hiromu Kutsumi, Yukihiro Koretsune, Takashi Yoshiyama, Marinus J. Blok, Nicholas Boddicker, Joan Brunet, Elizabeth S. Burnside, Mariarosaria Calvello, Ian Campbell, Sock Hoai Chan, Fei Chen, Jian Bang Chiang, Anna Coppa, Laura Cortesi, Ana Crujeiras-González, Consortium CZECANCA, Marianna Borecka, Marta Cerna, Milena Hovhannisyan, Sandra Jelinkova, Petr Nehasil, Lenka Foretova, Eva Machackova, Vera Krutilkova, Spiros Tavandzis, Leona Cerna, Stepan Chvojka, Monika Koudova, Alena Puchmajerova, Ondrej Havranek, Jan Novotny, Kamila Vesela, Michal Vocka, Lucie Hruskova, Renata Michalovska, Denisa Schwetzova, Zdenka Vlckova, Monika Cerna, Marketa Hejnalova, Nikol Jedlickova, Ivan Subrt, Tomas Zavoral, Marcela Kosarova, Gabriela Vacinova, Maria Janikova, Romana Kratochvilova, Vaclava Curtisova, Radek Vrtel, Ondrej Scheinost, Petra Duskova, Viktor Stranecky, Kim De Leeneer, Robin De Putter, Allison DePersia, Lisa Devereux, Susan Domchek, Anna Efremidis, Christoph Engel, Corinna Ernst, D. Gareth R. Evans, Lidia Feliubadaló, Florentia Fostira, Olivia Fuentes-Ríos, Encarna B. Gómez-García, Sara González, Christopher Haiman, Thomas van Overeem Hansen, Jan Hauke, James Hodge, Chunling Hu, Hongyan Huang, Nur Diana Binte Ishak, Yusuke Iwasaki, Irene Konstantopoulou, Peter Kraft, James Lacey, Conxi Lázaro, Na Li, Weng Khong Lim, Sara Lindstrom, Adriana Lori, Elana Martinez, Alexandra Martins, Koichi Matsuda, Giuseppe Matullo, Simone McInerny, Kyriaki Michailidou, Marco Montagna, Alvaro N.A. Monteiro, Luigi Mori, Katherine Nathanson, Susan L. Neuhausen, Heli Nevanlinna, Janet E. Olson, Julie Palmer, Barbara Pasini, Alpa Patel, Maria Piane, Bruce Poppe, Paolo Radice, Alessandra Renieri, Nicoletta Resta, Marcy E. Richardson, Toon Rosseel, Kathryn J. Ruddy, Marta Santamariña, Elizabeth Santana Dos Santos, Lauren Teras, Amanda E. Toland, Amy Trentham-Dietz, Celine M. Vachon, Alexander E. Volk, Nana Weber-Lassalle, Jeffrey N. Weitzel, Lisa Wiesmuller, Stacey Winham, Siddhartha Yadav, Drakoulis Yannoukakos, Song Yao, Valentina Zampiga, Magnus Zethoven, Ze Wen Zhang, Tomas Zima, Amanda B. Spurdle, Ana Vega, Maria Rossing, Jesús Del Valle, Arcangela De Nicolo, Eric Hahnen, Kathleen B.M. Claes, Joanne Ngeow, Yukihide Momozawa, Paul A. James, Fergus J. Couch, Libor Macurek, Zdenek Kleibl

**Affiliations:** 1 Laboratory of Cancer Cell Biology, Institute of Molecular Genetics of the Czech Academy of Sciences, Prague, Czech Republic.; 2 Institute of Medical Biochemistry and Laboratory Diagnostics, First Faculty of Medicine, Charles University and General University Hospital in Prague, Prague, Czech Republic.; 3 Institute of Biology and Medical Genetics, First Faculty of Medicine, Charles University and General University Hospital in Prague, Prague, Czech Republic.; 4 Department of Pathophysiology, First Faculty of Medicine, Charles University, Prague, Czech Republic.; 5 A.C. Camargo Cancer Center and Oncology Center, Hospital Sirio-Libanes, Sao Paulo, Brazil.; 6 Department of Cancer Prevention & Control, Roswell Park Cancer Center, Buffalo, New York.; 7 WCHS Inc., Baltimore, Maryland.; 8 Human Molecular Genetics Laboratory, INRaSTES, National Center for Scientific Research "Demokritos," Athens, Greece.; 9 Department of Breast Medical Oncology, University of Texas MD Anderson Cancer Center, Houston, Texas.; 10 Division of Biostatistics, Institute for Health and Equity, and Cancer Center, Medical College of Wisconsin, Milwaukee, Wisconsin.; 11 WHI, USA.; 12 Slone Epidemiology Center, Boston University, Boston, Massachusetts.; 13 Center for Genomic Medicine, Copenhagen University Hospital, Copenhagen, Denmark.; 14 Institute of Medical Science, The University of Tokyo, Tokyo, Japan.; 15 Department of Clinical Genetics, Maastricht University Medical Centre, Maastricht, the Netherlands.; 16 Department of Quantitative Health Sciences, Mayo Clinic, Rochester, Minnesota.; 17 CARRIERS, USA.; 18 Centro de Investigación Biomédica en Red de Cáncer (CIBERONC), Madrid, Spain.; 19 Hereditary Cancer Program, Catalan Institute of Oncology, IDIBELL-IGTP-IDIBGI, L'Hospitalet, Barcelona, Spain.; 20 School of Medicine and Public Health, University of Wisconsin, Madison, Wisconsin.; 21 WWHS, Charlotte, North Carolina.; 22 Division of Cancer Prevention and Genetics, IEO, European Institute of Oncology, IRCCS, Milan, Italy.; 23 Cancer Genomics Laboratory, Peter MacCallum Cancer Centre, Melbourne, Australia.; 24 Sir Peter MacCallum Department of Oncology, University of Melbourne, Melbourne, Australia.; 25 Cancer Genetics Service, National Cancer Centre, Singapore, Singapore.; 26 Lee Kong Chian School of Medicine, Nanyang Technological University, Singapore, Singapore.; 27 Keck School of Medicine, University of Southern California, Los Angeles, California.; 28 MEC, USA.; 29 Department of Experimental Medicine, Sapienza University of Rome, Rome, Italy.; 30 Department of Oncology and Haematology, Modena University Hospital, Modena, Italy.; 31 Fundacion Publica Galega de Medicina Xenomica, Santiago de Compostela, Spain.; 32 Instituto de Investigación Sanitaria de Santiago de Compostela (IDIS), Santiago de Compostela, Spain.; 33 Center for Medical Genetics, Ghent University and Ghent University Hospital, Ghent, Belgium.; 34 Center for Medical Genetics, NorthShore University Health System, Evanston, Illinois.; 35 Lifepool, Peter MacCallum Cancer Centre, Melbourne, Australia.; 36 Department of Medicine, Perelman School of Medicine, University of Pennsylvania, Philadelphia, Pennsylvania.; 37 Clinical Cancer Genetics and Family Consultants, CLINICAGENE, Athens Medical Center, Athens, Greece.; 38 Institute for Medical Informatics, Statistics and Epidemiology, University of Leipzig, Leipzig, Germany.; 39 Center for Familial Breast and Ovarian Cancer, Center for Integrated Oncology (CIO), University of Cologne, Faculty of Medicine and University Hospital Cologne, Cologne, Germany.; 40 Manchester Centre for Genomic Medicine, Division of Evolution and Genomic Sciences, University of Manchester, Manchester, United Kingdom.; 41 Molecular Diagnostics Laboratory, INRaSTES, National Center for Scientific Research "Demokritos," Athens, Greece.; 42 Department of Clinical Genetics, Copenhagen University Hospital, Copenhagen, Denmark.; 43 Department of Clinical Medicine, Faculty of Health and Medical Sciences, Copenhagen University, Copenhagen, Denmark.; 44 Behavioral and Epidemiology Research Group, American Cancer Society, Atlanta, Georgia.; 45 CPS3, Kennesaw, Georgia.; 46 Department of Laboratory Medicine and Pathology, Mayo Clinic, Rochester, Minnesota.; 47 T.H. Chan School of Public Health, Harvard University, Cambridge, Massachusetts.; 48 NHS, Reston, Virginia.; 49 Laboratory for Genotyping Development, RIKEN Center for Integrative Medical Sciences, Yokohama, Japan.; 50 Beckman Research Institute, City of Hope Cancer Center, Duarte, California.; 51 CTS, USA.; 52 Duke-NUS Medical School, Singapore, Singapore.; 53 Department of Epidemiology, University of Washington, Seattle, Washington.; 54 American Cancer Society, Atlanta, Georgia.; 55 Department of Family Medicine and Public Health, University of California San Diego, San Diego, California.; 56 Inserm UMR1245, UNIROUEN, Normandy Centre for Genomic and Personalized Medicine, Normandie University, Rouen, France.; 57 Institute of Medical Science, The University of Tokyo, Tokyo, Japan.; 58 Department of Medical Sciences, University of Turin, Turin, Italy.; 59 Parkville Familial Cancer Centre, Peter MacCallum Cancer Centre, and Royal Melbourne Hospital, Melbourne, Australia.; 60 Biostatistics Unit, The Cyprus Institute of Neurology and Genetics, Nicosia, Cyprus.; 61 Immunology and Molecular Oncology Unit, Veneto Institute of Oncology, Padua, Italy.; 62 Cancer Epidemiology Program, Division of Population Sciences, H. Lee Moffitt Cancer Center & Research Institute, Tampa, Florida.; 63 Endocrine and Metabolic Disease Unit, ASST Spedali Civili of Brescia, Brescia, Italia.; 64 Department of Clinical and Experimental Sciences, University of Brescia, Brescia, Italy.; 65 Department of Population Sciences, Beckman Research Institute of City of Hope, Duarte, California.; 66 Department of Obstetrics and Gynecology, Helsinki University Hospital and University of Helsinki, Helsinki, Finland.; 67 MCBCS, USA.; 68 Department of Population Science, American Cancer Society, Atlanta, Georgia.; 69 CPS-II, USA.; 70 Department of Clinical and Molecular Medicine, Sapienza University of Rome, Rome, Italy.; 71 Department of Experimental Oncology, Molecular Bases of Genetic Risk and Genetic Testing Unit, Fondazione IRCCS Istituto Nazionale dei Tumori, Milan, Italy.; 72 Medical genetics, University of Siena, Siena, Italy.; 73 Department of Precision and Regenerative Medicine and Ionian Area, Medical Genetics Unit, University of Bari, Bari, Italy.; 74 Ambry Genetics, Aliso Viejo, California.; 75 Department of Oncology, Mayo Clinic, Rochester, Minnesota.; 76 Centro de Investigación en Red de Enfermedades Raras (CIBERER), Santiago de Compostela, Spain.; 77 Department of Cancer Biology & Genetics, Comprehensive Cancer Center, The Ohio State University, Columbus, Ohio.; 78 University of Wisconsin, Madison, Wisconsin.; 79 Mayo Clinic, Rochester, Minnesota.; 80 MMHS, USA.; 81 Institute of Human Genetics, University Medical Center Hamburg-Eppendorf, Hamburg, Germany.; 82 Natera Inc., Duarte, California.; 83 Department of Obstetrics and Gynecology, Ulm University, Ulm, Germany.; 84 Department Quantitative Sciences, Mayo Clinic, Rochester, Minnesota.; 85 Department of Medical Oncology, Mayo Clinic, Rochester, Minnesota.; 86 Roswell Park Comprehensive Cancer Center, Buffalo, New York.; 87 Biosciences Laboratory, IRCCS Istituto Romagnolo per lo Studio dei Tumori (IRST) "Dino Amadori," Meldola, Italy.; 88 Population Health Program, QIMR Berghofer Medical Research Institute, Brisbane, Australia.; 89 Center for Omics Sciences, IRCCS San Raffaele Scientific Institute, Milan, Italy.

## Abstract

**Purpose:**

Germline pathogenic variants in *CHEK2* confer moderately elevated breast cancer risk (odds ratio, OR ∼ 2.5), qualifying carriers for enhanced breast cancer screening. Besides pathogenic variants, dozens of missense *CHEK2* variants of uncertain significance (VUS) have been identified, hampering the clinical utility of germline genetic testing (GGT).

**Experimental Design:**

We collected 460 *CHEK2* missense VUS identified by the ENIGMA consortium in 15 countries. Their functional characterization was performed using *CHEK2*-complementation assays quantifying KAP1 phosphorylation and CHK2 autophosphorylation in human RPE1–*CHEK2*-knockout cells. Concordant results in both functional assays were used to categorize *CHEK2* VUS from 12 ENIGMA case–control datasets, including 73,048 female patients with breast cancer and 88,658 ethnicity-matched controls.

**Results:**

A total of 430/460 VUS were successfully analyzed, of which 340 (79.1%) were concordant in both functional assays and categorized as functionally impaired (*N* = 102), functionally intermediate (*N* = 12), or functionally wild-type (WT)–like (*N* = 226). We then examined their association with breast cancer risk in the case–control analysis. The OR and 95% CI (confidence intervals) for carriers of functionally impaired, intermediate, and WT-like variants were 2.83 (95% CI, 2.35–3.41), 1.57 (95% CI, 1.41–1.75), and 1.19 (95% CI, 1.08–1.31), respectively. The meta-analysis of population-specific datasets showed similar results.

**Conclusions:**

We determined the functional consequences for the majority of *CHEK2* missense VUS found in patients with breast cancer (3,660/4,436; 82.5%). Carriers of functionally impaired missense variants accounted for 0.5% of patients with breast cancer and were associated with a moderate risk similar to that of truncating *CHEK2* variants. In contrast, 2.2% of all patients with breast cancer carried functionally wild-type/intermediate missense variants with no clinically relevant breast cancer risk in heterozygous carriers.

**Translational Relevance**

Protein-truncating germline variants in the *CHEK2* gene confer a moderate breast cancer risk; however, the association of missense variants with breast cancer risk remains unknown. Thus, the majority of missense variants are clinically inconclusive variants of uncertain significance (VUS). We analyzed 430 *CHEK2* missense VUS identified during a routine germline genetic testing of patients with cancer in 15 countries. Using two parallel functional assays, the functional analysis concordantly categorized 340/430 variants identified in 3,660 (82.5%) out of 4,436 carriers found in 73,048 female patients with breast cancer and 88,658 controls from 10 countries. Subsequent case–control analysis showed that only carriers of functionally impaired missense variants (0.5% of all patients with breast cancer) were associated with a clinically significant, moderate breast cancer risk (OR, 2.83; 95% confidence interval, 2.35–3.41), comparable with the risk for the protein-truncating variants. Our results will allow a conclusive interpretation for the majority of *CHEK2* germline missense variants identified in patients with breast cancer from different ethnicities worldwide.

## Introduction

The checkpoint kinase 2 gene (*CHEK2*) codes for nuclear serine/threonine protein kinase CHK2 phosphorylating numerous intracellular proteins in response to DNA damage and several other stress signals (1). Since its discovery in 1999, recurrent germline *CHEK2* variants have been associated with breast cancer predisposition (2–4). In addition, subsequent studies indicated increased risk of other malignancies, including prostate, kidney, thyroid, and colon cancers (5, 6).

The prevalence of germline *CHEK2* variants differs among patients with breast cancer worldwide (6). Two recent large studies demonstrated that *CHEK2* is the second most frequently altered breast cancer predisposition gene among patients of European descent, surpassed by *BRCA2* and followed by *BRCA1* in frequency of germline pathogenic variants (7, 8). However, to high breast cancer risk associated with alterations in *BRCA1* or *BRCA2*, both studies confirmed association of germline pathogenic *CHEK2* variants with moderate breast cancer risk with odds ratio (OR) 2.5 and a cumulative lifetime breast cancer risk of 25%–30%.

Because the increased breast cancer risk, *CHEK2* analysis has become a routine component of germline gene panels for identification of individuals at risk (9). Although c.1100delC or other *CHEK2* variants leading to protein truncations or aberrant pre-mRNA splicing are considered clearly pathogenic, the vast majority of *CHEK2* missense variants are clinically inconclusive (variants of uncertain significance, VUS). The presence of VUS impairs the clinical utility of diagnostic panel analysis and negatively influences patients’ perception of germline genetic testing (GGT; refs. 10–12). Moreover, the risk associated with germline missense variants in breast/ovarian cancer predisposition genes may differ from that in truncating variants (13–15).

To increase our understanding of the clinical relevance of *CHEK2* VUS, we collected missense variants reported by members of the international ENIGMA (Evidence-based Network for the Interpretation of Germline Mutant Alleles) consortium (16) and analyzed the variants by functional assays in a human cell line model (17). We then collected 12 case–control datasets from the ENIGMA consortium members, including 161,706 patients with breast cancer and population-matched controls from 10 countries, and examined the breast cancer risk for carriers of functionally stratified *CHEK2* missense variants.

## Materials and Methods

### Collection of *CHEK2* missense variants

The ENIGMA consortium members were requested to report all *CHEK2* missense variants (annotated using the NCBI reference sequence NM_007194.4) identified in patients with any cancer type that were analyzed by a routine GGT approved by local ethical committees. The variants were obtained between June 5, 2019 and August 24, 2022 and are listed in Supplementary Table S1.

### Datasets of patients with breast cancer and controls

**Figure 1.**
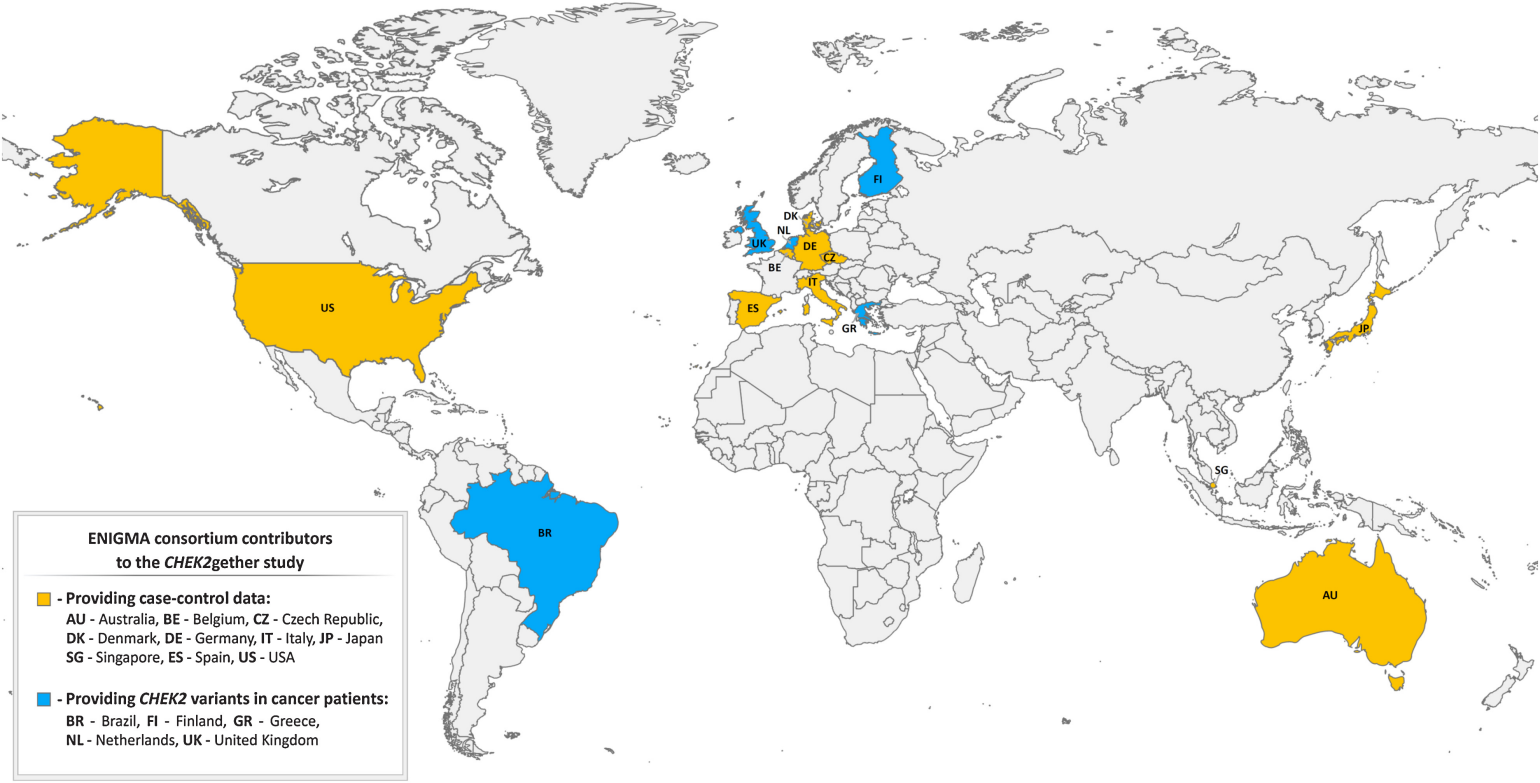
Geographical origin of analyzed *CHEK2* missense variants.

To estimate the breast cancer risk associated with *CHEK2* missense VUS, we collected 12 case–control datasets from 10 countries (Fig. 1) that included 73,048 breast cancer cases and 88,658 population-matched controls. The description of case–control datasets, including enrollment, ethical statements, ethnicity, and *CHEK2* analysis is provided in the Supplementary Table S2. Germline genetic analyses were conducted in accordance with the Declaration of Helsinki. Informed written consent for the participation in the studies, approved by institutional review boards, was obtained from each subject.

### Cell lines

Human non-transformed hTERT-RPE1 cells (ATCC CRL-4000, RRID: CVCL_4388) and their derivatives RPE1-CHEK2-KO cells with knockout (KO) of the endogenous *CHEK2* gene (17) were grown in DMEM media supplemented with 6% FBS (Gibco), penicillin (100 U/ml) and streptomycin (100 mg/mL). Cell lines were authenticated by using STR profiling and were regularly checked for the absence of mycoplasma infection using MycoAlert Plus reagent (Lonza; Cat# 75860–358). Where indicated, cells were exposed to ionizing radiation (IR; dose 5 Gy) generated by X-RAD 225XL (Precision).

### Plasmids

Coding sequence of the wild-type human *CHEK2* tagged at the N-terminus by FLAG sequence was cloned in frame into XhoI/EcoRI sites of pEGFP-C1 plasmid (Clontech; RRID: Addgene_165830). Subsequently, a panel of individual *CHEK2* mutants was generated by gene synthesis and was verified by a Sanger sequencing (Synbio Technologies).

### Antibodies

The following antibodies were used: Phospho-S473-KAP1 (BioLegend Cat# 654102, RRID: AB_2561782), phospho-S516-CHK2 (Cell Signaling Technology Cat# 2669, RRID: AB_330146), CHK2 (Abcam Cat# ab109413, RRID: AB_10863751), KAP1 (GeneTex Cat# GTX102227, RRID: AB_2037323), PCNA (Santa Cruz Biotechnology Cat# sc-56, RRID:AB_628110), goat anti-mouse (Thermo Fisher Scientific Cat# A-11004, RRID: AB_2534072), and goat anti-rabbit Alexa568 (Thermo Fisher Scientific Cat# A-11011, RRID: AB_143157).

### Immunofluorescence microscopy

RPE1–CHEK2-KO cells seeded on a glass bottom 96-well plate (Eppendorf) were transfected with an empty EGFP plasmid, wild-type pEGFP–CHEK2 or mutant pEGFP–CHEK2 using X-tremeGENE HP DNA Transfection Reagent (Roche). Cells were fixed with 4% paraformaldehyde 24 hours after transfection, permeabilized by 0.2% Triton X-100 in PBS for 7 minutes and blocked with 3% BSA in PBS at room temperature. Fixed cells were incubated with the KAP1-pS473 antibody or CHK2-pS516 antibody for 2 hours at room temperature, three times washed with PBS and incubated with the secondary antibodies and DAPI (Supplementary Methods). After washing with PBS, samples were mounted by Vectashield H-1000 and imaged using a ScanR microscope (Olympus) equipped with an ORCA-285 camera and 40×/1.3 NA objective. Mean intensities of the nuclear KAP1-pS473 (pKAP1) or CHK2-pS516 (pCHK2) signals were analyzed in GFP-positive cells using ScanR analysis software (Olympus).

### Functional categorization of *CHEK2* missense variants

For both assays, only pEGFP–CHEK2-transfected cells were selected. To avoid potential bias due to expression of the studied CHK2 isoforms at supraphysiological levels, only those cells expressing low levels of GFP, ranging within the linear pKAP1 to GFP signal for wt-plasmid transfected cells in each plate, were analyzed for the pKAP1 assay. In this analysis window, the enzymatic activity of each CHK2 variant was determined as an average value of the pKAP1/GFP ratio from >300 individual cells normalized to the wild-type CHK2. This step allowed to merge the outputs of individual plates from the screen.

For pCHK2 autophosphorylation assay, cells in the upper quartile of GFP signal for each variant were excluded, and only cells with GFP intensities lower than 200 A.U. were analyzed. Enzymatic activity of CHK2 variants was counted from >150 individual cells normalized to the wild-type CHK2 for each variant and determined as a “b” coefficient in a linear regression equation (*y* = a + bx; *x* corresponds to GFP, *y* corresponds to pCHK2 signals). All analyses were performed in RStudio version 4.2.1 (RRID: SCR_000432).

All relative enzymatic activity values higher than the lowest wild-type replica were considered functionally wild-type (WT)–like. All variants with enzymatic activity values lower than the highest catalytically dead control (the in-frame exon 7 deletion resulting in in frame deletion of 18 amino acids in kinase domain of CHK2, and EGFP, respectively) were considered functionally impaired. Variants scoring between the lowest wild-type and the highest impaired were categorized as “intermediate” (Supplementary Methods).

### Intracellular localization of CHK2 isoforms

The localization of all expressed CHK2 isoforms was assessed as the nuclear-to-cytoplasm ratio, calculated as the mean nuclear GFP signal intensity divided by the mean GFP signal intensity in a circular region outside the nucleus. Average of the nuclear-to-cytoplasm ratios from >300 individual cells were calculated for each analyzed variant (Supplementary Table S1). A set of 12 protein-truncating *CHEK2* variants lacking the nuclear localization signal (amino acids 515–522) served as controls with impaired nuclear localization.

### Statistical analyses

Variants for each “functional group” were combined to create a new variable used for the statistical testing in the form of a burden test. Variants were tested using the two-sided Fisher's exact test with *P* < 0.05 considered statistically significant. The meta-analysis of subgroups of populations was performed in R-studio 4.2.0 (RRID:SCR_000432 and RRID:SCR_001905) in library meta. The statistically significant *P* value (*P* < 0.05) and *I*^2^ > 75% indicated a heterogeneous sample. The analysis results were visualized using funnel and forest plots.

### Data availability

All frequency data of *CHEK2* VUS provided by the collaborating centers, kinase and localization assay data used to generate the figures' graphs and plots are provided in Supplementary Tables S1–S3. Raw data from high-content microscopy experiments are available upon request from the corresponding author.

## Results

### Identification of *CHEK2* missense variants

We and others have previously demonstrated that quantification of CHK2 kinase activity in human cells allows reliable scoring of the functional consequences of germline *CHEK2* missense variants (17, 18). The ensuing case–control analyses examining cancer risk in carriers of the functionally scoring variants showed promising results consistent with the functional categorization (17, 18). However, both studies were limited by the small number of *CHEK2* VUS analyzed or their selective ascertainment. Therefore, we aimed to perform a comprehensive analysis of unselected missense variants identified in routine GGT of cancer susceptibility. To this end, we collected 460 unique *CHEK2* germline missense VUS (Supplementary Table S1) identified in oncology patients by 20 members of the ENIGMA consortium from 15 countries (Fig. 1).

### Validation of cell-based assays for detection of CHK2 activity

**Figure 2.**
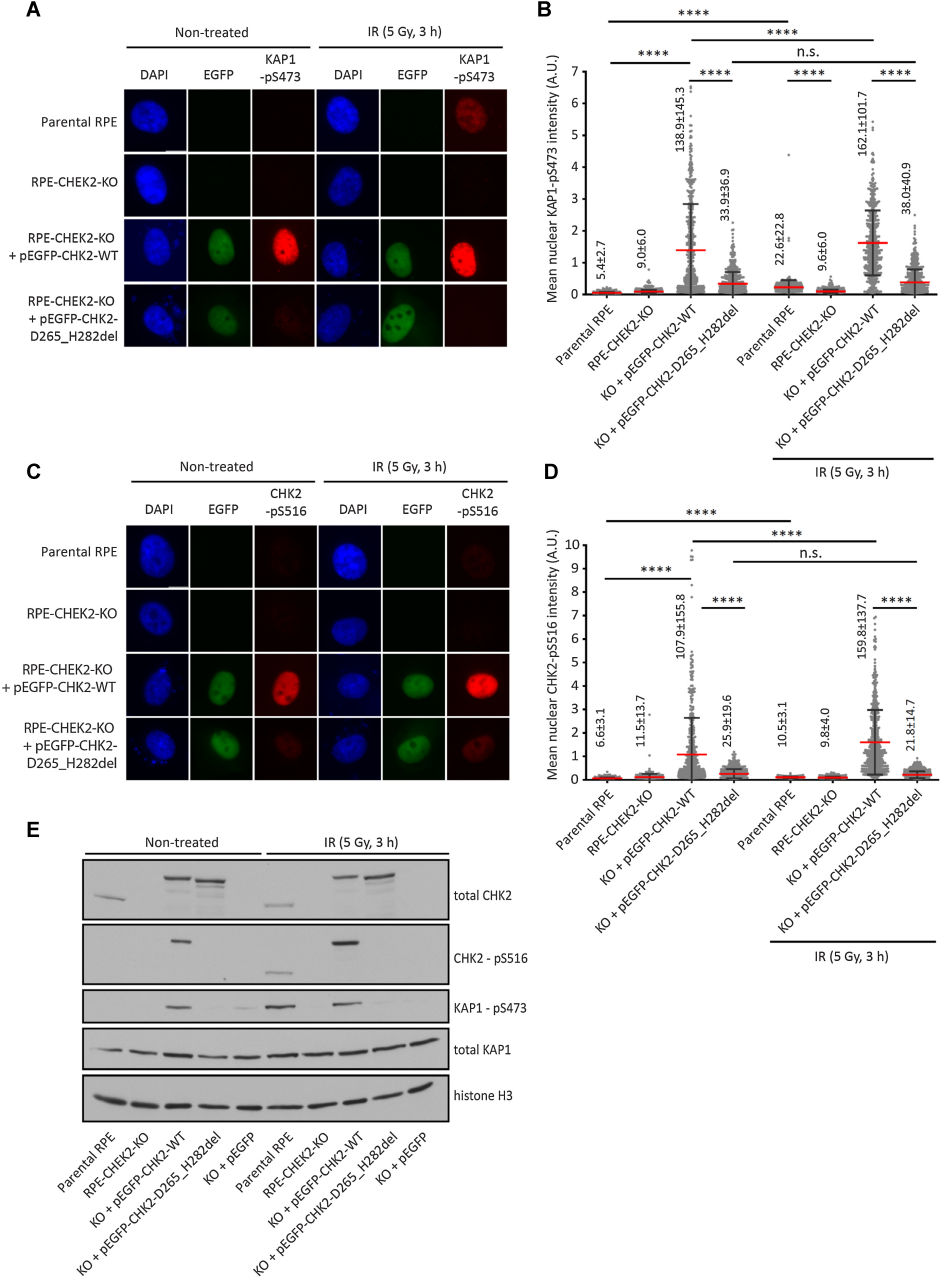
Validation of KAP1-pS473 and CHK2-pS516 antibodies. **A,** Parental RPE, RPE1–CHEK2-KO cells or RPE1–CHEK2-KO cells transfected with the wild-type or mutant pEGFP–CHEK2 were left untreated or were exposed to ionizing radiation (5 Gy, 3 hours). After fixation, cells were probed with KAP1-pS473 antibody. Representative images are shown. **B,** Quantification of **A**. The mean nuclear intensity of the KAP1-pS473 signal is plotted. Each dot represents one cell; more than 300 cells were analyzed. Red line, error bars and numbers indicate mean ± SDs. Statistical significance was evaluated by the Mann–Whitney test (****, *P* < 0.0001). A representative experiment is shown from two independent replicates. **C,** Cells were grown and treated as in **A** and were probed with CHK2-pS516 antibody. Representative images are shown. **D,** Quantification of **C**. The mean nuclear intensity of the CHK2-pS516 signal is plotted. Each dot represents one cell; more than 300 cells were analyzed. Red line, error bars and numbers indicate mean ± SDs. Statistical significance was evaluated by the Mann–Whitney test (****, *P* < 0.0001). A representative experiment is shown from two independent replicates. **E,** Cells were grown and treated as in A. Whole-cell lysates were analyzed by immunoblotting with indicated antibodies.

To improve functional characterization of the identified *CHEK2* VUS, we established two complementary functional assays for quantification of the catalytic activity of CHK2 in human, diploid, non-transformed RPE1 cells. We used previously described RPE1–*CHEK2*-KO cells with inactivated *CHEK2* and transiently transfected them with plasmids coding for EGFP-tagged *CHEK2* variants (17). Using high-content microscopy, we quantified the phosphorylation of the KRAB-associated protein 1 (KAP1) at S473, which is an established substrate of CHK2 (refs. 17, 19; Fig. 2A and B). We observed a low level of KAP1-pS473 in parental RPE1 cells, and the signal significantly increased after exposure of cells to IR, which is an activator of the ATM/CHK2 pathway (Fig. 2B). In contrast, we observed only background KAP1-pS473 signal in RPE1–*CHEK2*-KO cells and it was not responsive to IR (Fig. 2B). Upon transfection of the wild-type EGFP-CHK2, we observed a high nuclear KAP1-pS473 signal that further increased after exposure of cells to IR (Fig. 2B). Importantly, transfection of the catalytically dead EGFP-CHK2 p.D265_H282del variant did not increase the KAP1-pS473 signal, confirming specificity of the assay (Fig. 2B). Using the same approach, we determined the level of CHK2 autophosphorylation at S516 (refs. 20–23; Fig. 2C and D). The CHK2-pS516 signal of was low at endogenous levels of CHK2; nevertheless, a clearly detectable signal was observed in cells transfected with the wild-type EGFP-CHK2 and it was further increased upon exposure of cells to IR (Fig. 2D). In contrast, CHK2-pS516 signal was significantly lower in cells transfected with the catalytically dead CHK2, and it did not respond to IR (Fig. 2D). Similar results were obtained using detection of KAP1-pS473 and CHK2-pS516 by immunoblotting (Fig. 2E). We conclude that the nuclear KAP1-pS473 and CHK2-pS516 signal corresponds to the activity of EGFP-CHK2 transfected to RPE1–*CHEK2*-KO cells. Although the transfected EGFP-CHK2 was expressed at slightly higher levels than endogenous CHK2, phosphorylation of the substrates did not reach saturation as further increase of the signal was observed upon exposure to IR. We also noted that transfection of EGFP-CHK2 yielded a strong and reproducible KAP1-pS473 and CHK2-pS516 signal in basal conditions, and therefore we performed the functional screening of CHK2 VUS without exposing cells to IR.

### Functional assessment of *CHEK2* missense variants

Kinase activity analyses quantified phosphorylation of KAP1 (KAP1 assay) and autophosphorylation of CHK2 (CHK2 assay) using a high-content immunofluorescence microscopy that also allowed assessment of the intracellular localization of analyzed variants. Both assays were successfully performed for 430/460 (93.5%) variants (Supplementary Table S1). The remaining 30 variants were excluded from the analysis because the variants affected the first/last two coding nucleotides in an exon, and thus we cannot exclude that their functional consequence may result in aberrant splicing rather than in an amino acid change (16 variants) or due to poor expression (12 variants) or poor growth (2 variants) in RPE1 cells.

**Figure 3.**
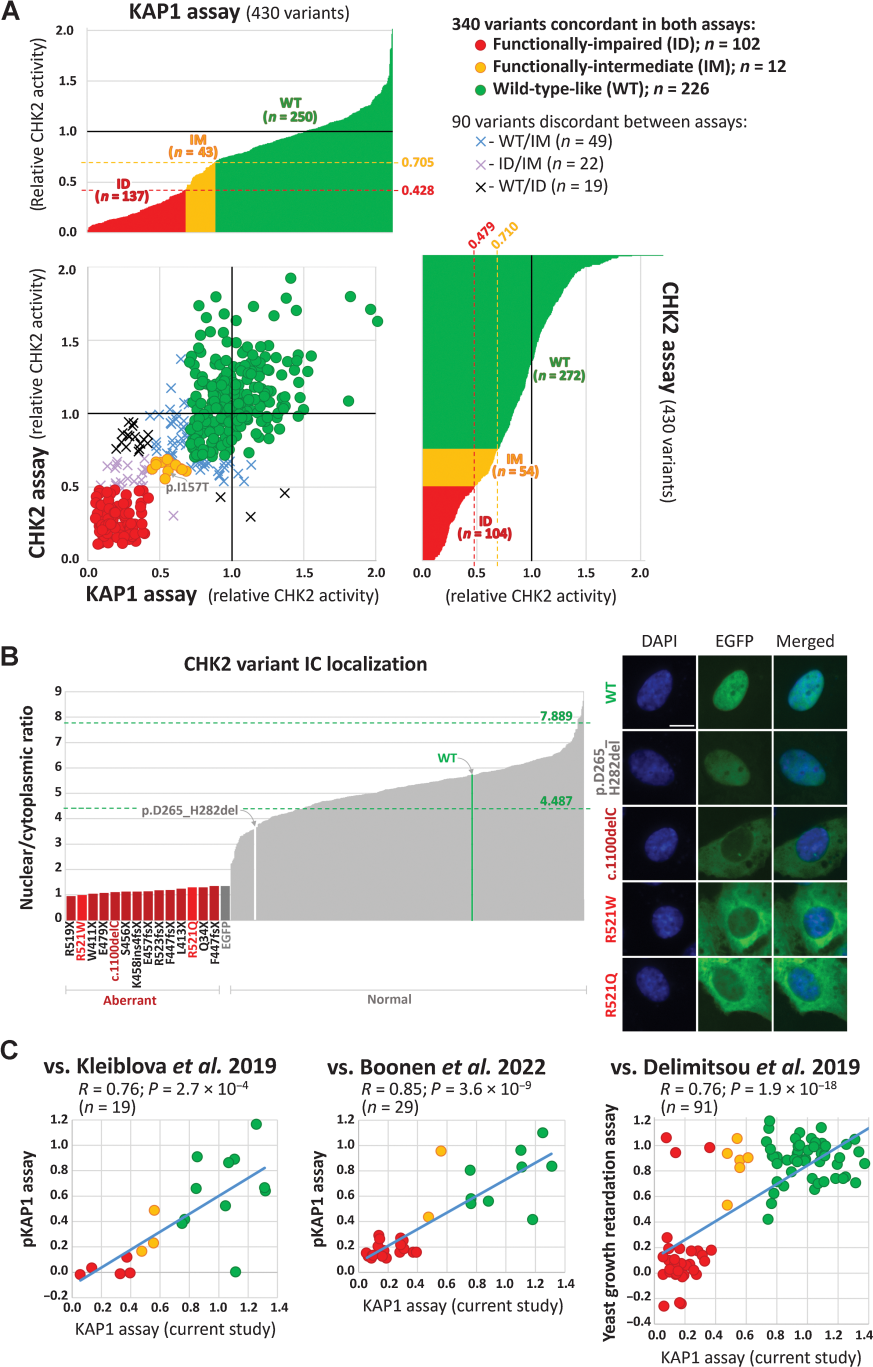
Kinase KAP1 and CHK2 assays (**A**). The bar graphs show results of kinase assays for 430 *CHEK2* missense variants. In both assays, variants with normalized relative CHK2 activity (mean WT-activity = 1) exceeding that of the weakest signal of WT replicas (not shown) were categorized functionally WT-like, variants with normalized signal intensity lower than the strongest signal for any of kinase-dead/empty EGFP vector controls (in-frame exon 7 deletion–p.D265_H282del; not shown) were categorized as functionally impaired. Variants with normalized CHK2 activities between these ranges were categorized functionally intermediate (0.428–0.705 and 0.479–0.710 for KAP1 and CHK2 assay, respectively; indicated by red and yellow dashed lines). Scatterplot combines results from both assays showing 340 concordant (circles) and 90 discordant (crosses) variants. The nuclear-to-cytoplasmic ratio (**B**) bar graph (left) displays all missense variants and a set of protein-truncating *CHEK2* variants (dark red bars at left, zoomed part of the graph). The missense variants, p.R521W and p.R521Q, with an aberrant localization are highlighted as bright-red bars; the arrows denote WT (green bar) and catalytically-dead in-frame p.D265_H282del variant (white bar). The highest and lowest mean nuclear/cytoplasmic ratio values from all WT replicates are indicated by green dashed lines. Of all missense variants analyzed by ScanR microscopy, only codon 521 alterations revealed aberrant intracellular localization with intense cytoplasmic positivity (right), reminiscent of mislocalization of the c.1100delC (p.T367fsX; size bar, 10 μm) variant. In comparison, the in-frame deletion p.D265_H282del revealed normal intranuclear accumulation, similar to WT. **C,** Scatter plots depicting correlations between assays performed in this study and previous analyses of *CHEK2* VUS. Studies of Kleiblova *et al.* (17) and Boonen et al. (18) used phosphorylation of KAP1 as a functional readout whereas the study of Delimitsou *et al.* (25) used a yeast growth retardation assay. The dots are colored according to the results of the KAP1 assay in this study (red, impaired; yellow, intermediate; green, wild-type–like). Blue line represents linear regression, R, correlation coefficient; *P*, *P* value. The scatter plot does not show the p.Arg512Trp variant classified by Boonen et al. as intermediate with impaired nuclear localization in our localization assay.

**Figure 4.**
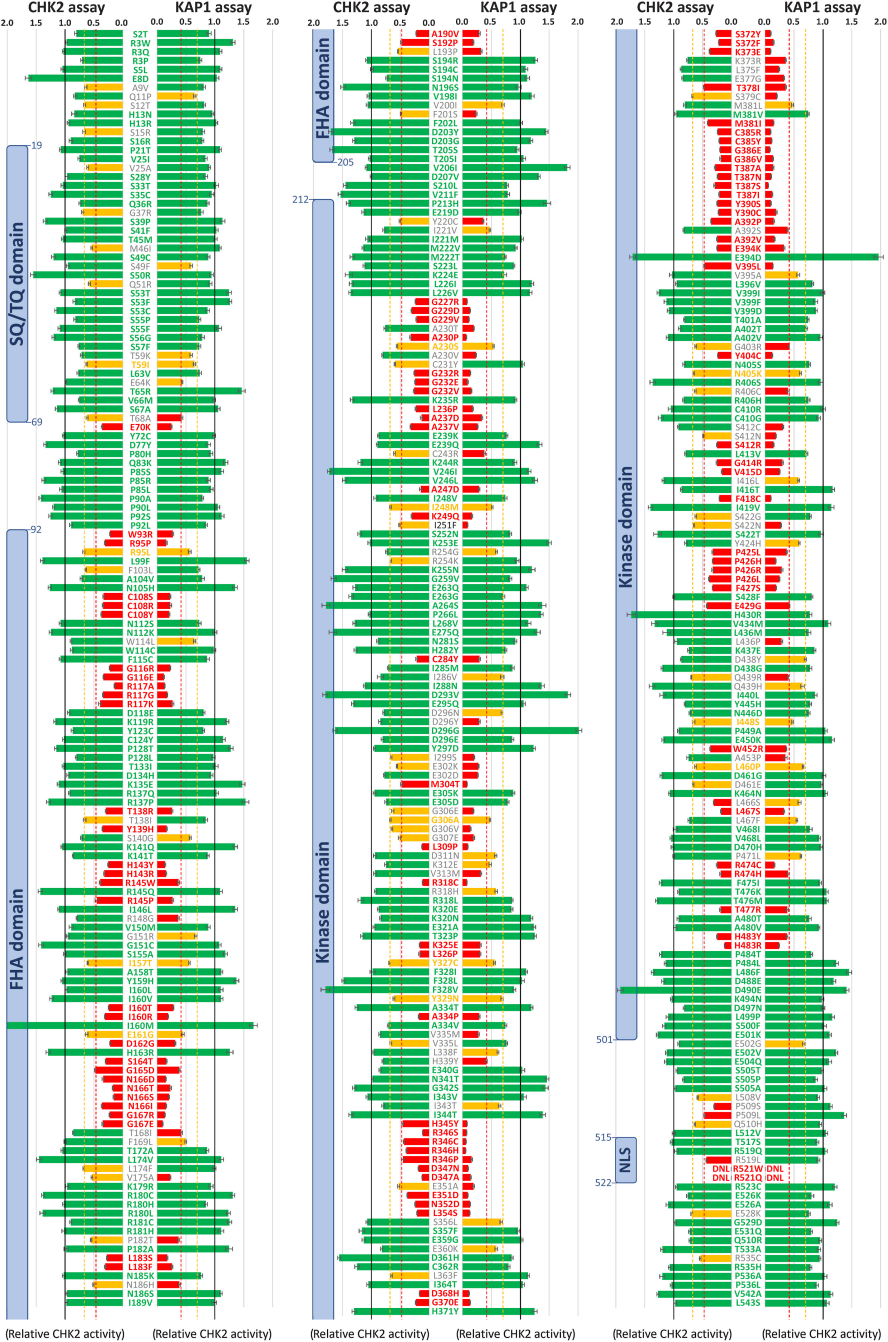
Results of KAP1 and CHK2 kinase assays for 430 successfully analyzed missense *CHEK2* variants (shown as an average relative CHK2 kinase activity). Bars are colored as functionally WT-like (green), intermediate (IM; yellow), and impaired (ID; red), respectively, with thresholds for IM variants (0.428 and 0.479) and ID variants (0.705 and 0.710) for KAP1 and CHK2 assays, respectively (dashed lines). Error bars represent standard errors of mean. Color/gray letters for protein variants indicate concordant/discordant functional assays result, respectively. Blue boxes denote conserved CHK2 domains. DNL, variants that do not localize into the nucleus.

The functional categorization in KAP1 and CHK2 assays were in accord for 340/430 (79.1%) successfully analyzed variants (Fig. 3A). Two thirds (226/340; 66.5%) of concordant variants scored functionally wild–type-like, whereas 12/340 (3.5%) and 102/340 (30.0%) scored functionally intermediate and functionally impaired, respectively. Among the 102 functionally impaired variants, we also included two variants, p.R521W and p.R521Q (scored WT/intermediate and intermediate/WT in kinase assays, respectively), which were the only analyzed missense variants with severely impaired nuclear localization (Fig. 3B). It is noteworthy that the most common c.470C>T (p.I157T) variant scored intermediate in both kinase assays that is consistent with KAP1 assay performed previously (17) and with its low (clinically unimportant) but statistically significant association with breast cancer risk (OR∼1.5) documented in heterozygous p.I157T carriers (24). Discordant categorization between KAP1 and CHK2 assays was observed for 90/430 (20.9%) variants with the WT/intermediate (49 variants) being the most common. A visualization of 430 successfully analyzed *CHEK2* missense variants is provided in Fig. 4, particular values for kinase assays and the nuclear-to-cytoplasmic ratio describing an intracellular localization are shown in Supplementary Table S1.

From 340 missense VUS concordant in kinase assays, 19 and 30 overlapped with variants analyzed previously using KAP1 assay by Kleiblova and colleagues (17) and Boonen and colleagues (18), respectively, and 91 overlapped with VUS analyzed by Delimitsou and colleagues (25) using a yeast growth retardation assay. A significant correlation between the KAP1 assay and the results of all three previous studies (Fig. 3C) demonstrates reliability of the functional assessment. Comparison of functional data for 340 variants concordantly categorized in our assays that were analyzed in aforementioned studies is provided in the Supplementary Table S1.

### Functionally categorized *CHEK2* missense variants and female breast cancer risk

To explore how variants categorized by our functional assay results associated with female breast cancer risk, we used 12 case–control datasets (Supplementary Table S2) provided by ENIGMA consortium members from 10 countries (Fig. 1). The majority of the 161,706 individuals (patients with breast cancer and unaffected controls) were of European descent (*n* = 117,877; 73.0%). Individuals of Asian (*n* = 33,535; 20.7%), African-American (*n* = 8,942; 5.5%), and other races/ethnicities (*n* = 799; 0.8%) were less frequent. Ascertainment for patient subgroups were heterogeneous, including family/hospital based (40,801; 55.9%) and unselected (32,247; 44.1%) female patients with breast cancer.

Among all 161,706 individuals, there were 4,436 carriers (2.7%) of 377 unique *CHEK2* missense VUS analyzed in functional assays. The majority (3,660/4,436; 82.5%) of VUS carriers were individuals carrying some of 272 variants that were categorized as concordant in both functional assays (Supplementary Table S1). Remaining 776 individuals were excluded, including 721 (16.3%) carriers of 78 variants discordantly categorized by kinase assays, 31 (0.7%) carriers of 13 variants suspected to interfere with pre-mRNA splicing, and 24 (0.5%) carriers of 14 variants that failed in the functional analysis. The baseline frequency of all 4,436 *CHEK2* VUS carriers was significantly higher in patients over controls [3.4% vs. 2.3%; OR, 1.52; 95% confidence interval (CI), 1.43–1.61] setting a low but significant background breast cancer risk. Increased proportion of variant carriers in cases over controls was maintained also for 3,660 carriers of concordantly categorized variants (2.8% vs. 1.9%; OR, 1.50; 95% CI, 1.40–1.60) with similar background risk (Supplementary Tables S1 and S3).

**Figure 5.**
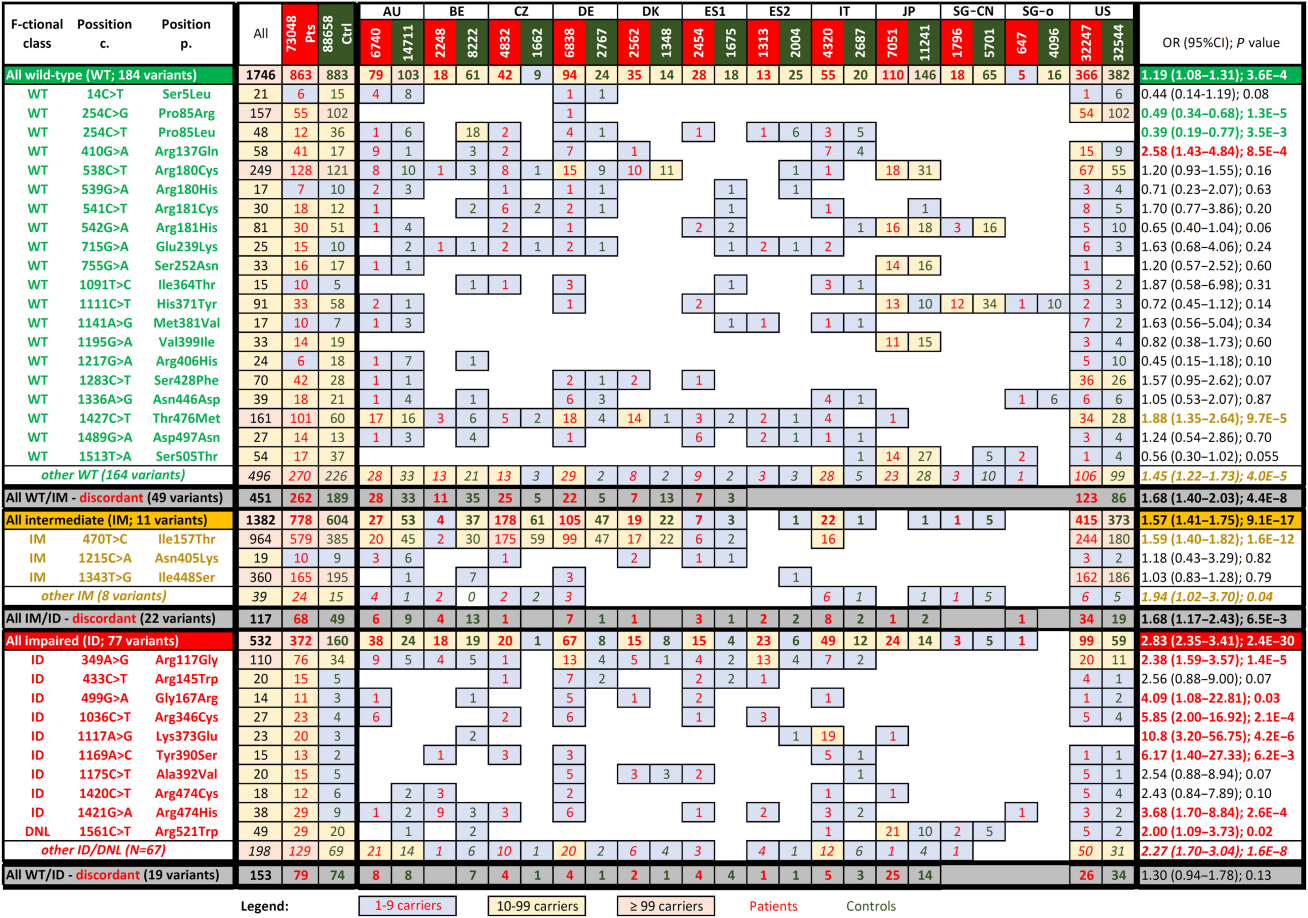
Presence of analyzed *CHEK2* missense variants categorized according to the functional assays in patients with breast cancer (BC pts; red numbers) and matched controls (dark green numbers). The association with breast cancer risk (odds ratio; OR) were calculated for prevalent variants having ≥10 carriers among patients or controls, respectively. Colors of the numbers in the last column highlight significant association with moderate-or-higher risk (red; OR > 2), low risk (OR < 2), protective variants (green) or variants without significant impact on breast cancer risk (black). Gray rows display variants that were discordant in the kinase assays. DNL, variants that do not localize into the nucleus.

First, we analyzed association with the breast cancer risks for the functionally characterized categories. In the burden analysis, the statistically significant OR gradually increased from 1.19 (95% CI, 1.08–1.31) to 1.57 (95% CI, 1.41–1.75), and 2.83 (95% CI, 2.35–3.41) for variants functionally characterized as WT-like, intermediate and impaired, respectively (Fig. 5). The non-overlapping 95% CIs indicate the reliable functional characterization discriminating between the risk categories.

The results of the subsequent case–control analysis of individual prevalent variants (identified in ≥10 carriers in patients or controls; Fig. 5) showed that the association with breast cancer differed from the functional categorization for two WT-like variants, reaching a significant moderate risk for p.R137Q (OR, 2.58; 95% CI, 1.43–4.84) and a modestly elevated risk for p.T476M (OR, 1.88; 95% CI, 1.35–2.64), respectively. Other WT-like variants were not associated with significantly increased risk or exerted a protective effect (p.P85R and p.P85L). The risk of functionally intermediate variants was predominantly influenced by p.I157T, as this variant accounted for 964/1382 (69.8%) carriers in the intermediate category. None of other individual variants from the functionally intermediate group were significantly associated with breast cancer risk. Among variants categorized as functionally impaired, 7/10 variants were individually significantly associated with breast cancer and the proportion of carriers among cases outnumbered those among controls for the remaining three variants.

In addition, we performed the burden case–control analysis for variants with discordant results in the kinase assays (Fig. 5) and also for variants affecting border exon sites, which are suspected to affect splicing. The latter category (not shown in the Fig. 5) included 20 carriers in patients and 11 in controls and was associated with increased breast cancer risk (OR, 2.21; 95% CI, 1.06–4.61; *P* = 0.03).

### Meta-analyses of functionally categorized population datasets

**Figure 6.**
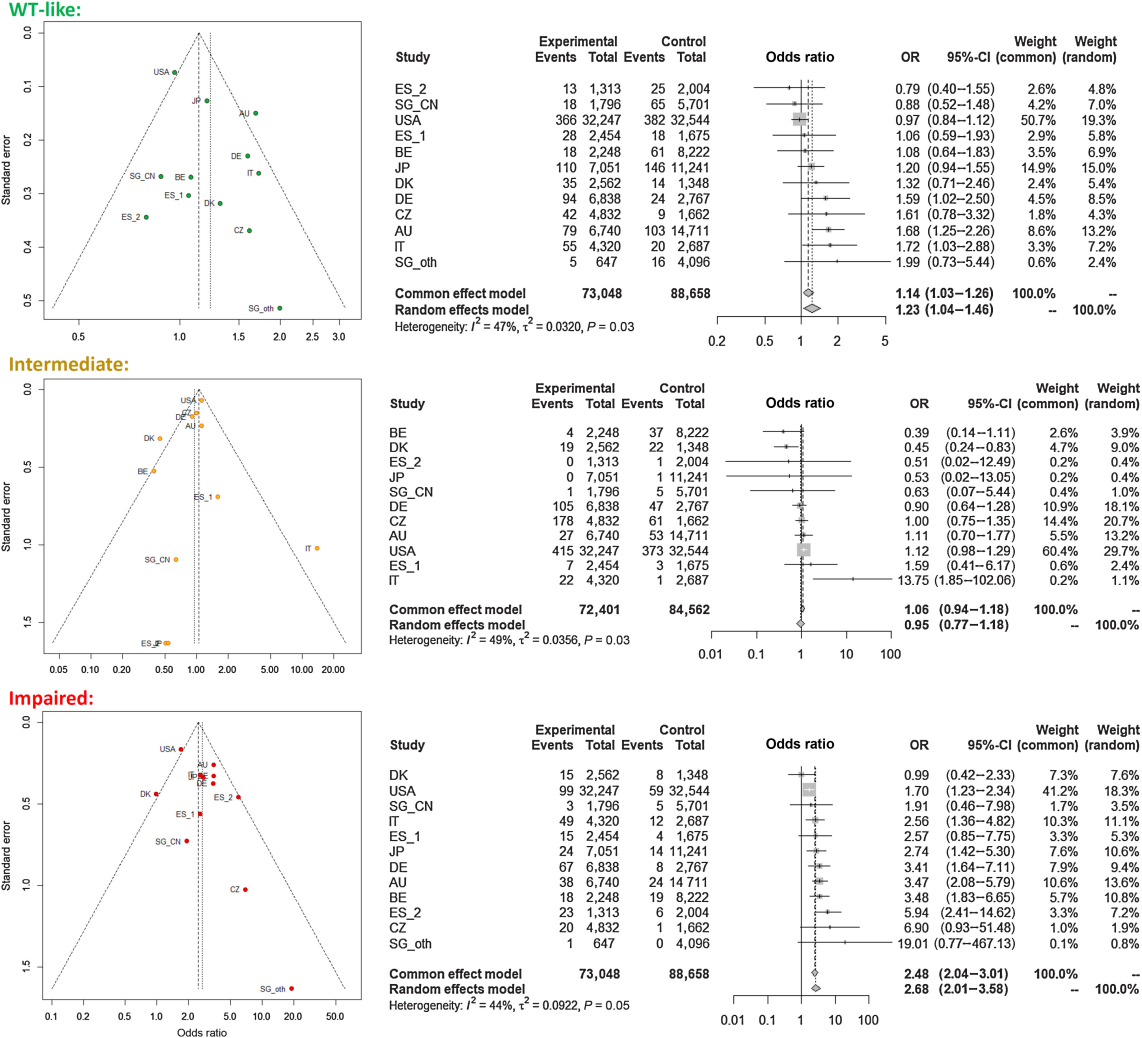
Funnel plot (left) and forest plots (right) for individual datasets of breast cancer cases and controls from 12 datasets (10 countries) stratified according to the functional categorization.

We performed the meta-analysis to assess the magnitude of the risk in the groups of *CHEK2* VUS carriers categorized according to the results of our functional assays (Fig. 6). The *P* values of <0.05 indicated substantial variability for the datasets in VUS functionally characterized as WT-like and intermediate. The variability was lower for the functionally impaired category retaining a significant moderate risk (OR, 2.68; 95% CI, 2.01–3.58 for random effect model) based on significantly increased risks in 7/12 individual population datasets. The risk for WT-like variants was marginally statistically significant but close to one and thus clinically irrelevant. The non-significant risk of the functionally intermediate group was affected by variable prevalence of the p.I157T variant, which was enriched in control populations from Belgium and Denmark but rare in Italian controls. The results of meta-analysis revealed large variability in case–control datasets but confirmed a low breast cancer risk in carriers of WT-like and intermediate variants, and a clinically considerable and significant breast cancer risk in carriers of variants that were categorized as functionally impaired.

## Discussion

GGT of the *CHEK2* gene yields appreciable frequencies of pathogenic or likely pathogenic (P/LP) germline variants in breast cancer or other patients with cancer (6, 26). On the other hand, genetic testing has also revealed a large number of missense variants that are mostly classified as VUS, hindering its clinical utility. This is illustrated by review of recent (January, 2023) ClinVar database data, registering 593 unique frameshift/nonsense/splice-site *CHEK2* alterations of which 566 (95.4%) received conclusive classification [564 P/LP and 2 benign/likely-benign (B/LB)]. In contrast, only 15/1,497 (1.0%) *CHEK2* missense variants in ClinVar had non-conflicting conclusive classification (including 6 P/LP and 9 B/LB). Analysis of gnomAD data indicated that the overall frequencies of stop-gain/frameshift/splice-site (1.65%) and missense (1.87%) *CHEK2* germline variants were comparable.

A large, international, case–control study of the Breast Cancer Association Consortium (BCAC; ref. 7) identified rare *CHEK2* missense variants (i.e., variants with a population frequency of <0.001) in 2.0% patients with breast cancer and 1.4% controls, indicating slightly increased association with breast cancer for missense variants as a group (OR, 1.43; 95% CI, 1.31–1.57). Frequency of *CHEK2* missense variants in our study was 3.4% in patients with breast cancer and 2.2% in controls (OR, 1.52; 95% CI, 1.43–1.61; Supplementary Table S3). When applying the rare variant definition from the BCAC study (i.e., excluding carriers of p.I157T, p.I448S, and p.R180C), missense variant frequency reduced to 2.2% in cases and 1.5% in controls and the association with breast cancer was comparable with that reported by BCAC (OR, 1.51; 95% CI, 1.40–1.62 for variants with frequency <0.001). This association indicates that in addition to functionally neutral variants, functionally impaired missense variants increasing breast cancer risk are enriched among patients with breast cancer. The clinical need urges for clear discrimination between the pathogenic and non-pathogenic variants as both may modify clinical management in their carriers (and relatives in case of pathogenic alterations).

The ACMG guidelines provide a generally adopted framework to standardize variant interpretation in clinical settings (27). However, additional credible methods for variant interpretation are required to effectively harness data from GGT. Validated functional assays have been considered as one of the most powerful tools to aid variant interpretation (28, 29). Our study is the largest and the most comprehensive functional analysis of real-world germline *CHEK2* missense VUS found in patients with various cancer diagnoses and in controls. Corroborated by the case–control analysis of variant groups stratified according to the results of our functional assays, it provides several meaningful insights:

First, the *CHEK2* functional analysis, based on a combination of KAP1/CHK2 assays with a high-content microscopy controlling the intracellular targeting of analyzed variant in human non-cancer cells, scored concordantly for 340 variants. This more than doubled the number of 160 *CHEK2* missense VUS categorized so-far (reviewed recently in ref. 14) using various approaches. In comparison with our previous functional study (17) describing the KAP1-based *CHEK2* analysis, we implemented improved calculations of relative KAP1-phosphorylation intensities normalized to CHK2 expression, added CHK2 autophosphorylation and localization assays in human cells and abandoned synthetic *in vitro* phosphorylation assays. Our current results showed a high correlation with the results from our previous KAP1 assay (17), the KAP1 assay by Boonen and colleagues (18) in mouse embryonic stem cells and also with yeast-based assay by Delimitsou and colleagues (25). To the yeast survival assays, our assays provide the assessment of CHK2 kinase activity in physiologic intracellular environment, including natural CHK2 substrates, inhibitors, and interactors.

Second, the burden case–control analysis allowed us to determine association of the functionally categorized missense variants with breast cancer. A clinically significant moderate association with breast cancer risk was observed for carriers of functionally impaired variant for both simplistic burden analysis (OR, 2.83; Fig. 5) and meta-analysis of individual population-specific datasets (OR, 2.68; Fig. 6). The burden case–control analysis of the most common truncating variant c.1100delC performed in our datasets revealed a similar association with breast cancer risk (OR, 2.73; 95% CI, 2.29–3.26; Supplementary Table S1). Comparable associations were noted in previous meta-analyses of c.1100delC in unselected breast cancer populations (OR, 2.75; 95% CI, 2.25–3.36 and OR, 2.89; 95% CI, 2.63–3.16; refs. 30, 31). Thus, we demonstrate that the breast cancer risk associated with functionally impaired *CHEK2* missense variants is comparable with the risk associated with P/LP truncating variants. In addition, the study conferred that carriers of functionally impaired missense variants account for 0.5% patients with breast cancer overall. This was substantially lower than 1.3% predicted by Dorling and colleagues (32) in BCAC data analyzed by *in silico* prediction tools. However, the proportion of carriers varied among national datasets in our study, being highest in European patients (0.9%) and less frequent in patients from the USA (0.3%) and patients from Japan and Singapore (0.2%). On the other hand, 2.2% of all patients with breast cancer in this study were carriers of missense variants categorized as WT-like or intermediate with clinically irrelevant breast cancer risk.

Third, the case–control analyses of individual variants largely corresponded to their functional categorization. The association of p.R117G (the most common variant from the functionally impaired class and the sixth most frequent variant in our dataset) with breast cancer risk (OR, 2.38; 95% CI, 1.58–3.68) was similar to the risk of the entire functionally impaired subgroup. A comparable risk (OR, 2.26; 95% CI, 1.29–3.95) for p.R117G was reported by Southey and colleagues (33) in the Collaborative Oncological Gene-environment Study and recently by Dorling and colleagues (ref. 32; OR, 2.69; 95% CI, 1.46–4.94) analyzing the BCAC data. Interestingly, the p.I157T has been categorized as functionally intermediate with modestly elevated association with breast cancer risk (OR, 1.59; 95% CI, 1.40–1.82), which was comparable with previously published p.I157T meta-analyses with OR ranging between 1.28 and 1.48 in unselected patients with breast cancer (6). Of note, case–control analyses and the functional characterization were discrepant for several variants. The category of functionally WT-like variants included mostly variants with OR∼1.0, but two variants, p.R137Q and p.T476M (fully functional in our kinase assays), showed elevated associations with breast cancer risk in case–control analyses (OR = 2.58 and OR = 1.88, respectively). Although both variants were frequent (the tenth and the fourth most common in our study, respectively), they were virtually absent in Asian populations, and present with large variability in populations of European ancestry (Fig. 5). They have been subjected to functional analyses previously. Although p.R137Q was described as neutral by Sodha and colleagues (34) and Bell and colleagues (35), the functional data for p.T476M were rather discrepant (14). Recent case–control analysis of large cohort of patients with cancer, including 250 carriers of p.T476M, revealed only a modest association with the breast cancer risk (OR, 1.35; 95% CI, 1.03–1.77; *P* = 0.03) and no risk of other analyzed cancers (36). In the functionally intermediate subgroup, we included the p.I448S variant, which was considered intermediate in the KAP1 assay in Boonen and colleagues (18) and also the yeast assay in Delimitsou and colleagues (25). Prevalent in the US data, the variant had OR∼1 in the case–control analysis and it is classified a B/LB in ClinVar. In the functionally impaired subgroup, 7/10 variants showed concordantly significantly increased OR>2 in case–control analysis. These partially conflicting results indicate that larger case–control studies will be required to refine the risks for individual rare VUS but for population-specific variants, case–control data from founder populations will be of particular importance. In addition, extended functional assays would help to determine the boundary between WT-like and intermediate categories more precisely; however, it should be emphasized that both subgroups have low or negligible clinical impact, at least for carriers of heterozygous variants. Individual-level case–control analyses and/or additional functional/splicing assays will remain important to inform classification of functionally intermediate/WT-like variants because some individual variants may impact CHK2 function via mechanisms not surveyed by our assays. However, taken together, the functional analysis will facilitate clinical classification of germline *CHEK2* missense VUS, in particular as evidence toward pathogenicity for variants concordantly categorized as functionally impaired.

Finally, the meta-analyses of functionally characterized categories indicated that although some case–control datasets were heterogeneous, meta-analyses findings corresponded to those from the burden analysis.

We are aware of several limitations of this study. Although we selected the cells with low CHK2 expression for the functional analysis of VUS, we cannot completely exclude that the assay could be affected by the expression of the studied CHK2 isoforms at supraphysiological levels; however, this concern is unlikely to affect the categorization of the functionally impaired variants. The carriers were identified by different genotyping approaches and variant reporting differed among contributing centers of ENIGMA members. The patient populations included hospital-based, high-risk, and unselected patients with breast cancer in individual datasets. This ascertainment bias could slightly overestimate the breast cancer risks for variant carriers. Because no other clinicopathological data were available, we could not analyze the associations of *CHEK2* variants with clinicopathological variables.

In conclusion, we functionally categorized a majority of germline *CHEK2* missense variants commonly occurring in patients with cancer and controls in various populations worldwide. The case–control analysis revealed that the breast cancer risk of functionally impaired germline variants is comparable with that of truncating *CHEK2* P/LP variants and suggested that the clinical management of (breast) cancer prevention in both groups of carriers should be similar (37). Moreover, the comprehensive functional classification will foster development of clinical interpretation guidelines for germline *CHEK2* variants and will allow exploration of the association of germline functionally impaired missense variants with other cancer types. The data may also serve as a predictive information for tailored anticancer therapy using PARP or immune checkpoint inhibitors in carriers of germline functionally impaired variants (38–40).

## Supplementary Material

Supplementary Methods 1 Detail description of the functional categorization of analyzed CHEK2 missense variants.

Table S1 List of all analyzed CHEK2 variants with results of KAP1/CHK2 kinase and localization assays and the results from recent previously published functional analyses of the CHEK2 VUS.

Table S2 Characteristics of 12 case-control datasets from the ENIGMA consortium partners.

Table S3 Frequencies of all reported germline CHEK2 variant carriers and carriers of variants concordantly categorized by functional our kinase assays in breast cancer patients and controls in 12 analyzed population datasets.
