# Supplementary material for: ENIGMA CHEK2gether Project: A Comprehensive Study Identifies Functionally Impaired CHEK2 Germline Missense Variants Associated with Increased Breast Cancer Risk
Source: Clin Cancer Res. 2023 Jul 13;29(16):3037–50. doi: 10.1158/1078-0432.CCR-23-0212 (PMC10425727; doi:10.1158/1078-0432.CCR-23-0212)
Supplement: Supplementary Methods 1 — Detail description of the functional categorization of analyzed CHEK2 missense variants. [file ccr-23-0212_supplementary_methods_1_suppsm_1.docx]

**ENIGMA *CHEK2*gether project: a comprehensive study identifies functionally-impaired *CHEK2* germline missense variants associated with increased breast cancer risk**

Lenka Stolarova, Petra Kleiblova, Petra Zemankova, Barbora Otahalova, Marketa Janatova, Jana Soukupova, Maria Isabel Achatz, Christine Ambrosone, Paraskevi Apostolou, Banu K. Arun, Paul Auer, Mollie Barnard, Birgitte Bertelsen, Biobank Japan, Marinus J. Blok, Nicholas Boddicker, Joan Brunet, Elizabeth S. Burnside, Mariarosaria Calvello, Ian Campbell, Sock Hoai Chan, Fei Chen, Jian Bang Chiang, Anna Coppa, Laura Cortesi, Ana Crujeiras-González, Consortium CZECANCA, Kim De Leeneer, Robin De Putter, Allison DePersia, Lisa Devereux, Susan Domchek, Anna Efremidis, Christoph Engel, Corinna Ernst, Gareth D. Evans, Lidia Feliubadaló, Florentia Fostira, Olivia Fuentes-Ríos, Encarna B. Gómez-García, Sara González, Christopher Haiman, Thomas van Overeem Hansen, Jan Hauke, James Hodge, Chunling Hu, Hongyan Huang, Nur Diana Binte Ishak, Yusuke Iwasaki, Irene Konstantopoulou, Peter Kraft, James Lacey, Conxi Lázaro, Na Li, Weng Khong Lim, Sara Lindstrom, Adriana Lori, Elana Martinez, Alexandra Martins, Koichi Matsuda, Giuseppe Matullo, Simone McInerny, Kyriaki Michailidou, Marco Montagna, Alvaro N.A. Monteiro, Luigi Mori, Katherine Nathanson, Susan L. Neuhausen, Heli Nevanlinna, Janet E. Olson, Julie Palmer, Barbara Pasini, Alpa Patel, Maria Piane, Bruce Poppe, Paolo Radice, Alessandra Renieri, Nicoletta Resta, Marcy E. Richardson, Toon Rosseel, Kathryn J. Ruddy, Marta Santamariña, Elizabeth Santana Dos Santos, Lauren Teras, Amanda E. Toland, Amy Trentham-Dietz, Celine M. Vachon, Alexander E. Volk, Nana Weber-Lassalle, Jeffrey N. Weitzel, Lisa Wiesmuller, Stacey Winham, Siddhartha Yadav, Drakoulis Yannoukakos, Song Yao, Valentina Zampiga, Magnus Zethoven, Ze Wen Zhang, Tomas Zima, Amanda B. Spurdle, Ana Vega, Maria C. Rossing, Jesús Del Valle, Arcangela De Nicolo, Eric Hahnen, Kathleen B. M. Claes, Joanne Ngeow, Yukihide Momozawa, Paul A. James, Fergus J. Couch, Libor Macurek, Zdenek Kleibl.

***Functional categorization of CHEK2 missense variants in details***

***Assay design***

The assays (outlined in the Supplementary Methods Figure 1) included analysis of a kinase activity of the tested *CHEK2* missense variants in RPE1-CHEK2-KO cells with knocked-out endogenous *CHEK2* gene derived from human non-transformed hTERT-RPE1 cells. RPE1-CHEK2-KO cells were seeded on a 96-well glass bottom plate and transfected with an empty EGFP plasmid, wild-type pEGFP-CHEK2, or mutant pEGFP-CHEK2. Each plate included 60 wells used for the analysis (leaving all outer wells of the 96-well plate empty to avoid edge effects), including 4-8 WT controls, 4-8 catalytically-dead controls, 2 non-transfected controls, and 40-48 analyzed *CHEK2* missense variants. Each missense variant was analyzed at least in two independent plates.

Cells were fixed 24 h after transfection, permeabilized and blocked. Fixed cells were incubated with a primary antibody, washed with PBS and incubated with the secondary antibodies (labeled by AlexaFluor568) and DAPI (for a nuclear staining). After washing with PBS, samples were mounted by Vectashield H-1000 and imaged using a ScanR microscope (Olympus). Mean intensities of the nuclear pKAP1-S473 signals in GFP-positive cells were analyzed using ScanR analysis software (Olympus).

Analysis of the kinase activity of tested *CHEK2* missense variants was performed using two targets of phosphorylation. The first assay quantified phosphorylation of the natural CHK2 kinase substrate KAP1 (normally expressed in RPE-1 cells) at its serine 473. The second assay quantified autophosphorylation at serine 516 in the C-terminus of CHK2 kinase.

| **Supplementary Methods Figure 1. Schematic representation of the functional assays scoring kinase activity of germline CHK2 isoforms.** The assays included analysis of phosphorylation of the natural CHK2 kinase substrate KAP1 at its serine 473. The KAP1 is expressed normally in RPE-1 cells (Figure 2E). The second assay involved quantification of autophosphorylation at serine 516 in C-terminus of CHK2 kinase. The analysis for both assays were similar in principle and differed by the use of detection antibody (described in details in the Methods section) and in some details during data processing (commented herein). |
| --- |
| 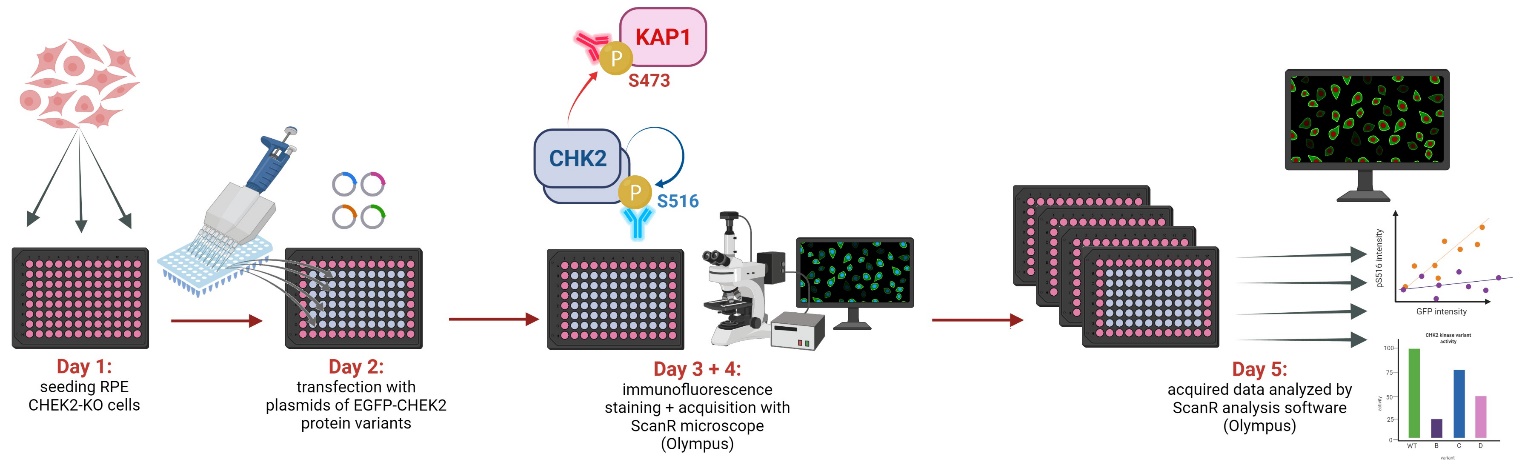 |

***KAP1-pS473 assay***

All analyzed cells were first determined to be the GFP-negative (non-transfected cells) or the GFP-positive (transfected cells). This step served for the elimination of non-transfected cells from the analysis and the threshold was estimated individually for each plate. The **GFP-positive cells** were considered to be those cells with GFP levels above a threshold of 4-16 arbitrary units (A.U.) in a high-content immunofluorescence microscopy (Supplementary Methods Figure 2; Supplementary Methods Table 1). This lower threshold was determined as a value that would be higher than the GFP signal of over 98% of the non-transfected cells (analyzed in each 96-well plate).

| **Supplementary Methods Figure 2.** **An example of data from a single 96-well plate (plate #13) depicting 32,767 GFP-positive cells from a selected high-content microscopy run.** In the example (A) of plate #13, the lower gating threshold (eliminating GFP-negative cells) was set at 7 A.U.; the displayed run (plate) contained a total of 32,767 GFP-positive cells, and the one with maximum GFP signal was 3106 A.U.). The gated analysis window (B) included in this experiment all cells in GFP interval between 7 – 85 A.U. which contained 20,566 low-GFP cells out of 32,767 (62.8%) GFP-positive cells. This gating step was performed to exclude cells expressing analyzed CHK2 variants in supraphysiological concentrations that may phosphorylate all naturally occurring KAP1 substrates even in in variants with reduced/partially impaired catalytic activity. The marks indicate individual GFP-positive cells: red diamonds describe catalytically dead controls, green dots WT-controls and blue diamonds all analyzed missense variants. |
| --- |
| 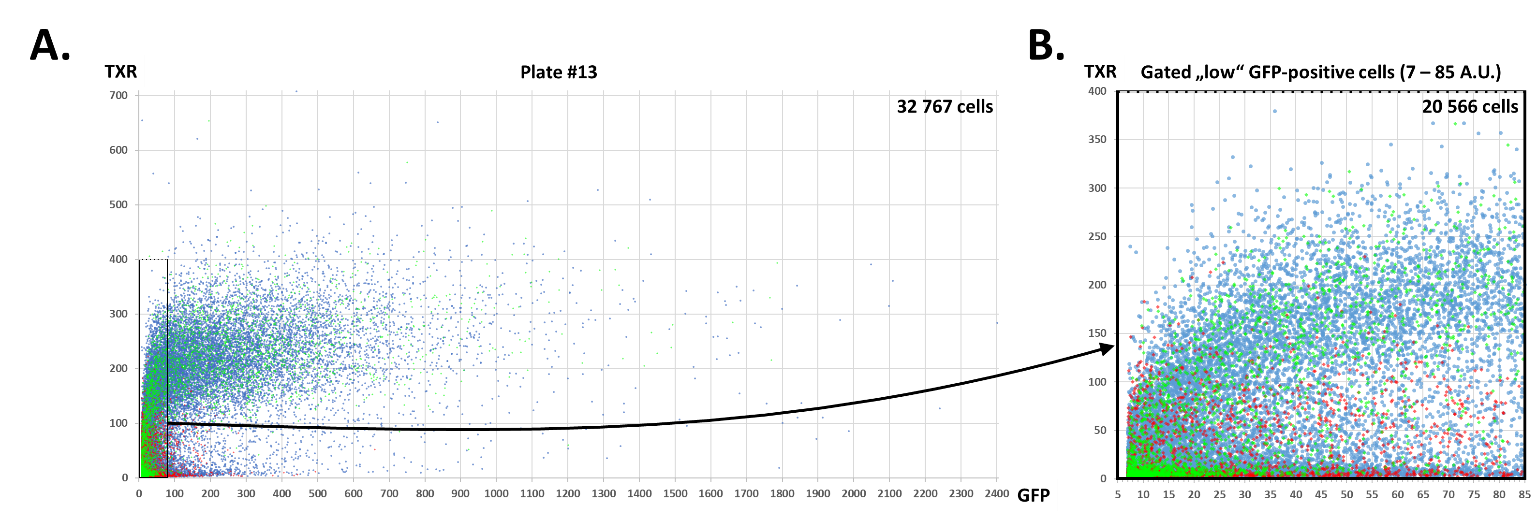 |

In a subsequent step, we determined an analysis GFP window in which the KAP1 phosphorylation signal (y-axis) increased quasilinearly (prior reaching the saturation phase of KAP1 phosphorylation) in GFP-positive WT cells and the number of cells was sufficient for the analysis. This **gating** step was performed for each individual plate to exclude the cells expressing the analyzed CHK2 variants at the supraphysiological concentrations. The upper gating threshold was determined from the analysis of all WT samples in a given run, tracing the mean of pKAP1-S473 signal increment in decimal GFP intervals, at a range starting from the lower gating limit of GFP-positive cells up to 600 A.U. (including the saturation phase of KAP1 phosphorylation; (Supplementary Methods Figure 3). The Supplementary Methods Table 1 provides the values of upper gating limits (in all analyzed 96-well plates used for variant categorization) not exceeding 200 A.U.

| **Supplementary Methods Figure 3. Setting the analysis window (from the plate #13;** Supplementary Methods Figure 2**).** The curves show mean values of pKAP1-S473 (y-axis; numbers indicate mean A.U.; red line) and the cell numbers (y-axis; numbers indicate number of cells; blue line) in decimal GFP intervals. The analysis window contained 2 096 (55.3%) low-GFP cells out of 3 789 GFP-positive cells (scored in the interval 7 – 600 A.U.) in WT replicas from the plate #13. |
| --- |
| 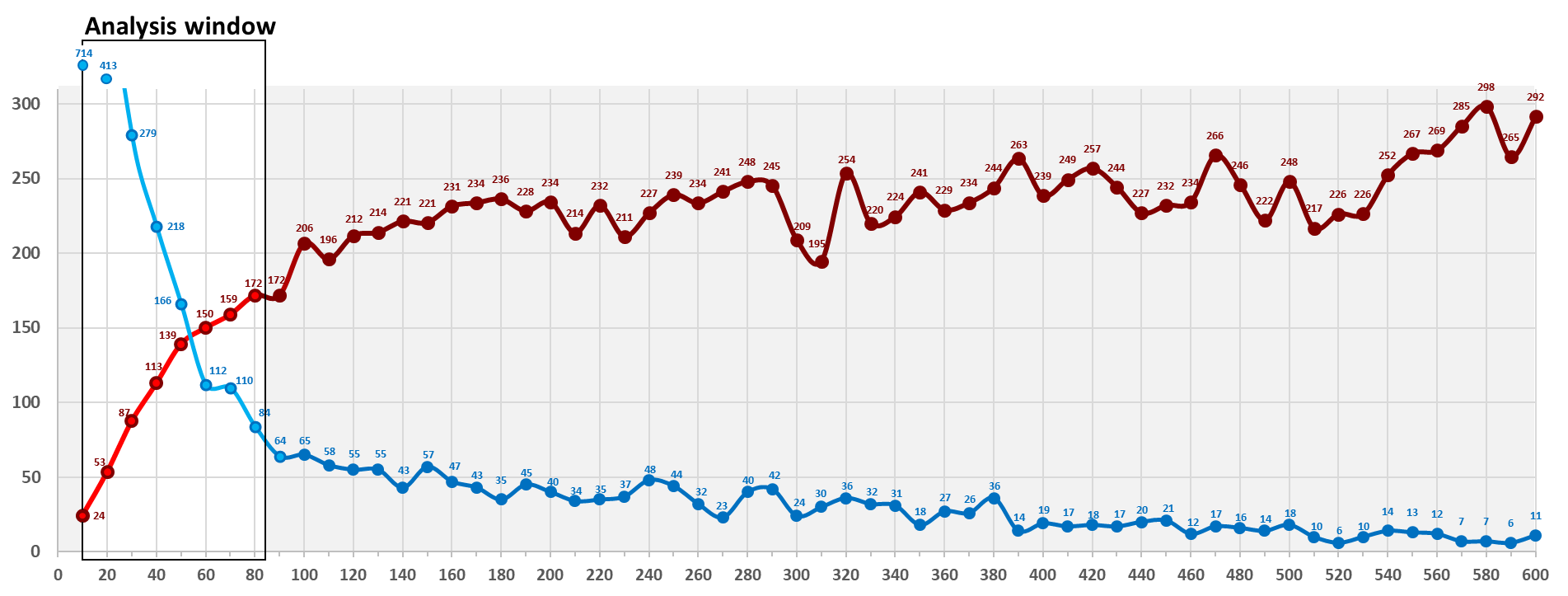 |

**Supplementary Methods Table 1.** The table describes parameters of analyses in all 25 plates included into the analysis of missense *CHEK2* variants. The parameters include number of GFP-positive cells, number and fraction (%) of cells that were used for quantification of KAP1 signal (gated cells), lower and upper gate limit used for quantification of kinase activity in linear phase and the maximal GFP value indicating that gated cells represent a “low” GFP-positive cell population (selected to analyze the CHK2 kinase activity in linear phase and also to prevent expression of analyzed variants in supraphysiological concentrations).

| **plate #** | **GFP-positive cells (N)** | **Gated**  **GFP-positive**  **cells (N)** | **Gated**  **GFP-positive**  **cells (%)** | **Lower gate GFP limit [A.U.]** | **Upper gate GFP limit [A.U.]** | **Maximal**  **GFP value**  **[A.U.]** |
| --- | --- | --- | --- | --- | --- | --- |
| 1 | 86 659 | 25 193 | 29 | 8 | 95 | 3 984 |
| 1r | 78 686 | 20 974 | 27 | 4 | 105 | 2 845 |
| 2 | 38 387 | 7 491 | 20 | 7 | 85 | 2 203 |
| 2r | 104 000 | 33 160 | 32 | 4 | 85 | 3 341 |
| 3 | 74 783 | 17 145 | 23 | 4 | 125 | 2 969 |
| 3r | 113 880 | 34 632 | 30 | 4 | 105 | 3 087 |
| 4 | 119 894 | 37 675 | 31 | 4 | 115 | 3 261 |
| 4r | 57 951 | 17 588 | 30 | 4 | 95 | 3 727 |
| 4r2 | 79 926 | 30 089 | 38 | 12 | 165 | 3 858 |
| 5 | 129 180 | 33 159 | 26 | 4 | 105 | 3 824 |
| 5r | 76 711 | 21 088 | 27 | 10 | 185 | 3 892 |
| 6 | 175 059 | 43 694 | 25 | 5 | 95 | 3 585 |
| 6r | 85 475 | 23 506 | 28 | 12 | 135 | 3 705 |
| 7 | 47 741 | 19 985 | 42 | 16 | 155 | 3 602 |
| 10 | 21 486 | 10 468 | 49 | 11 | 80 | 3 084 |
| 11 | 45 047 | 30 541 | 68 | 6 | 75 | 2 564 |
| 11r | 24 895 | 13 902 | 56 | 6 | 75 | 3 297 |
| 12 | 27 077 | 20 642 | 76 | 6 | 75 | 3 179 |
| 12r | 28 908 | 18 877 | 65 | 6 | 75 | 3 201 |
| 13 | 32 767 | 20 566 | 63 | 7 | 85 | 3 106 |
| 13r | 31 709 | 21 128 | 67 | 7 | 85 | 3 916 |
| 14 | 26 210 | 16 776 | 64 | 7 | 85 | 2 788 |
| 14r | 28 365 | 16 951 | 60 | 7 | 85 | 3 559 |
| 15 | 13 492 | 7 780 | 58 | 7 | 85 | 2 625 |
| 15r | 15 656 | 7 596 | 49 | 7 | 85 | 3 096 |
| *All analyzed cells* | *1 563 944* | *550 606* | *-* | *-* | *-* | *-* |

The gated intervals (analysis windows) were used for a subsequent analysis of individual variant in a given plate (Supplementary Methods Figure 4).

| **Supplementary Methods Figure 4. An example of the values from immunofluorescent microscopy in the analysis window (from the** Supplementary Methods Figure 2B**).** The graphs show GFP (x-axis) and pKAP1-S473 (y-axis) values of the two WT replicas (green doted frame), two kinase dead controls (red dotted frame), and six variants (blue dotted frame). The number of cells counted in the gated window is shown in the brackets. |
| --- |
| 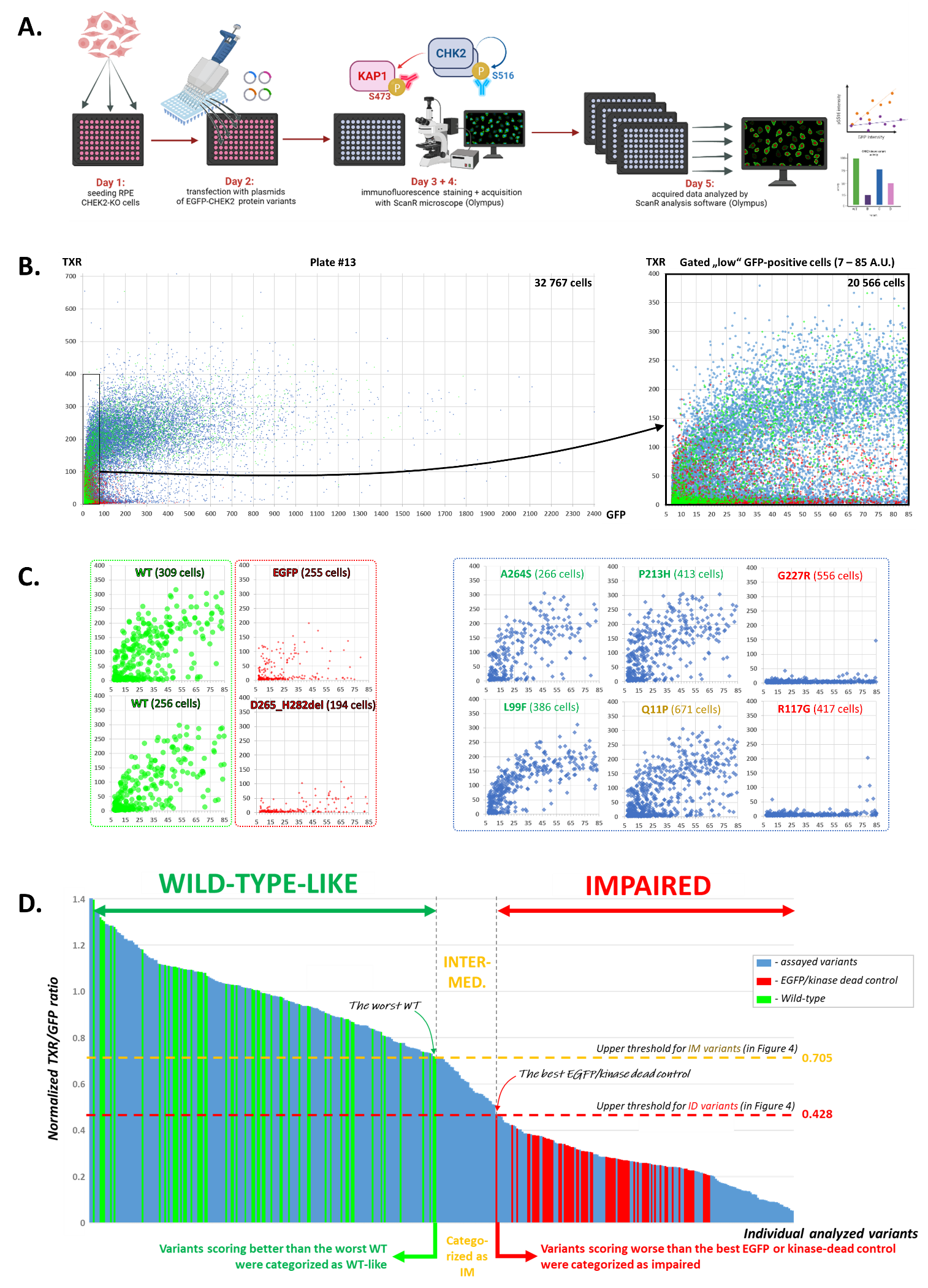 |

After determining the analysis window (Supplementary Methods Figure 3), we performed a **normalization** that allowed us to eliminate the inter-run variability of high-content immunofluorescence microscopy and to summarize the pKAP1‑S473/GFP signals of individual variants and cells from different runs thereafter. To this end, we first calculated the GFP and pKAP1-S473 coefficients, from mean GFP and pKAP1-S473 of all cells transfected with WT CHK2 controls within the gate in a given plate (i.e. from all wells containing WT replicas, regardless of their position in the plate). Thereafter, the GFP and pKAP1-S473 values of each cell were divided by these normalization coefficients. The normalization step allowed us to pool together individual cells with a particular variant from independent plates. The **enzymatic activity was determined** as the mean of normalized pKAP1-S473 /GFP values (± SD) for each variant tested.

***CHK2-pS516 assay***

Even when the CHK2-pS516 autophosphorylation assay is not limited by the substrate concentration as it is in the KAP1-pS473 assay, we excluded cells expressing analyzed CHK2 variants in supraphysiological concentrations.

The **GFP-positive cells** (the same estimation as in KAP1 assay) were used for the analysis. The upper-gating step was not introduced for individual plates (as in KAP1 assay), but was determined to 200 A.U., because the increased concentration of expressed CHK2 represented the increase of its substrate for autophosphorylation, at the same time. Subsequently, the intraplate normalization steps were performed by the same way as in KAP1 analysis. The **enzymatic activity of CHK2 variants** was normalized to the wild-type CHK2 for each variant and determined as a “b” coefficient in a linear regression equation (y = a + bx; where x corresponds to GFP, and y corresponds to pKAP1-S473 signals). All analyses were performed in RStudio version 4.2.1.

***Variant categorization***

Only variants having at least 300 and 150 cells from KAP1 and CHK2 assay, respectively, were considered for variant categorization. The numbers of individual cells analyzed in particular variants in both assays are shown in Supplementary Table S1 (excel file).

The individual analyzed variants (blue bars) were plotted according to their decreasing values of enzyme activity (i.e. normalized pKAP1-S473/GFP ratio values) including individual normalized values for all WT replicas (bright green bars) and all replicas of vectors expressing EGFP-only or kinase-dead controls (red bars). The variants with normalized values exceeding the weakest signal of WT replica were categorized functionally-WT-like (WT-like), variants with normalized signal intensity lower than the strongest signal for any of kinase-dead/empty EGFP vector controls were categorized as functionally-impaired (ID). Variants with normalized CHK2 activities between these ranges were categorized functionally-intermediate (IM). Thus, the upper threshold limit for IM variants in KAP1 assay is 0.705 and upper threshold limit for ID variants is 0.428. Similarly, these limits are 0.710 and 0.479 for the CHK2 autophosphorylation assay, respectively; (Supplementary Methods Figure 5).

| **Supplementary Methods Figure 5. A graph depicting the categorization of analyzed variants in KAP1 kinase assay in a single analysis pool** (an example includes data for 288 variants analyzed in 12 well plates). The normalized pKAP1-S473/GFP values of analyzed variants (blue bars) were plotted according to their decreasing values of enzyme activity (i.e. normalized pKAP1-S473/GFP ratio values) including individual normalized values for all WT replicas (bright green bars) and all replicas of vectors expressing EGFP-only or kinase-dead controls (red bars). The variants with normalized values exceeding the weakest signal of WT replica were categorized functionally-WT-like (WT-like), variants with normalized signal intensity lower than the strongest signal for any of kinase-dead/empty EGFP vector controls were categorized as functionally-impaired (ID). Variants with normalized CHK2 activities between these ranges were categorized functionally-intermediate (IM). Thus, the upper threshold limit for IM variants in KAP1 assay is 0.705 and upper threshold limit for ID variants is 0.428. Similarly, these limits are 0.710 and 0.479 for the CHK2 autophosphorylation assay, respectively; (Figure 4). |
| --- |
| 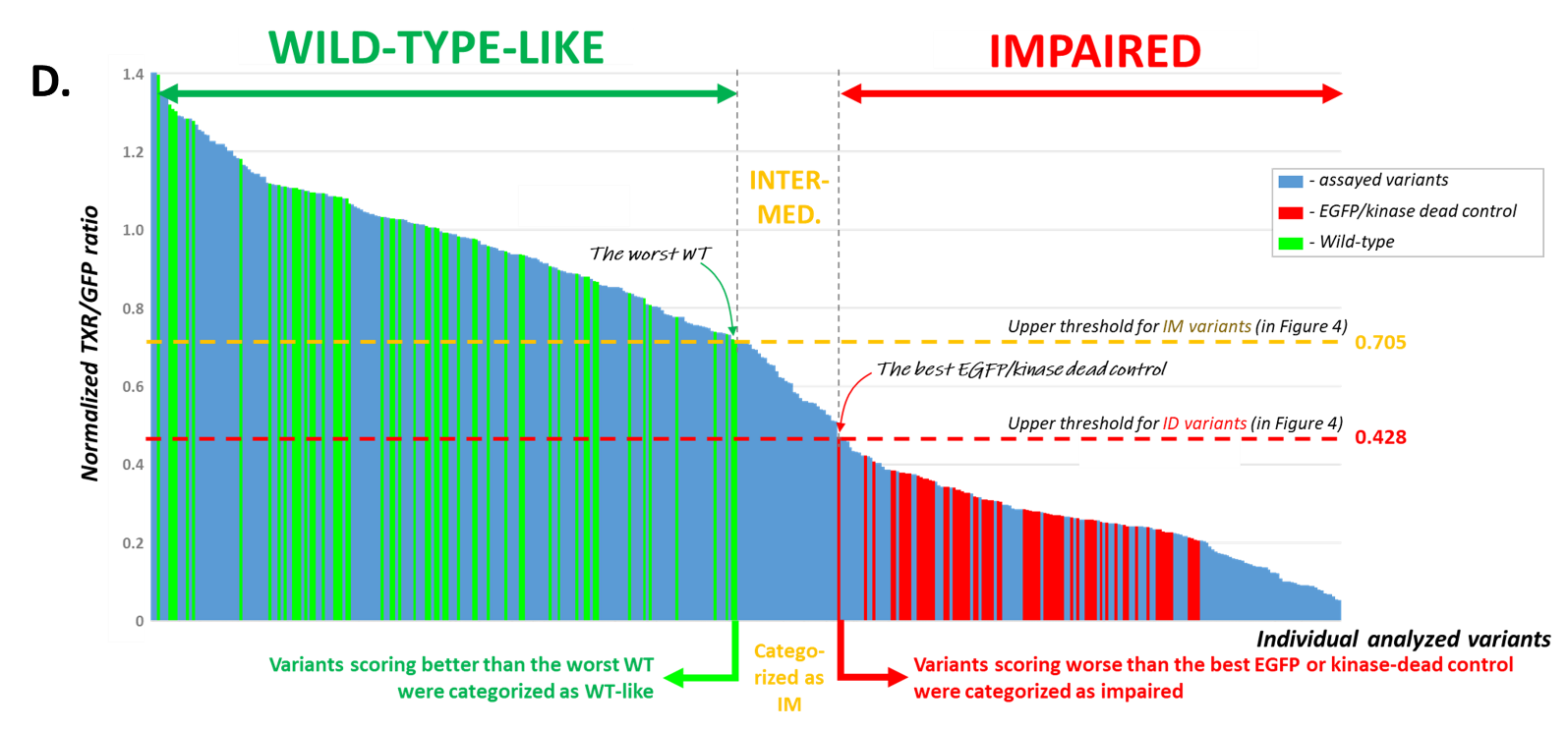 |
